# Supplementary material for: Antifungal Activity of the Extract of a Macroalgae, Gracilariopsis persica, against Four Plant Pathogenic Fungi
Source: Plants (Basel). 2021 Aug 26;10(9):1781. doi: 10.3390/plants10091781 (PMC8467150; doi:10.3390/plants10091781)
Supplement: Supplementary file 1 [file plants-10-01781-s001.zip › plants-1335982-supplementary.pdf]

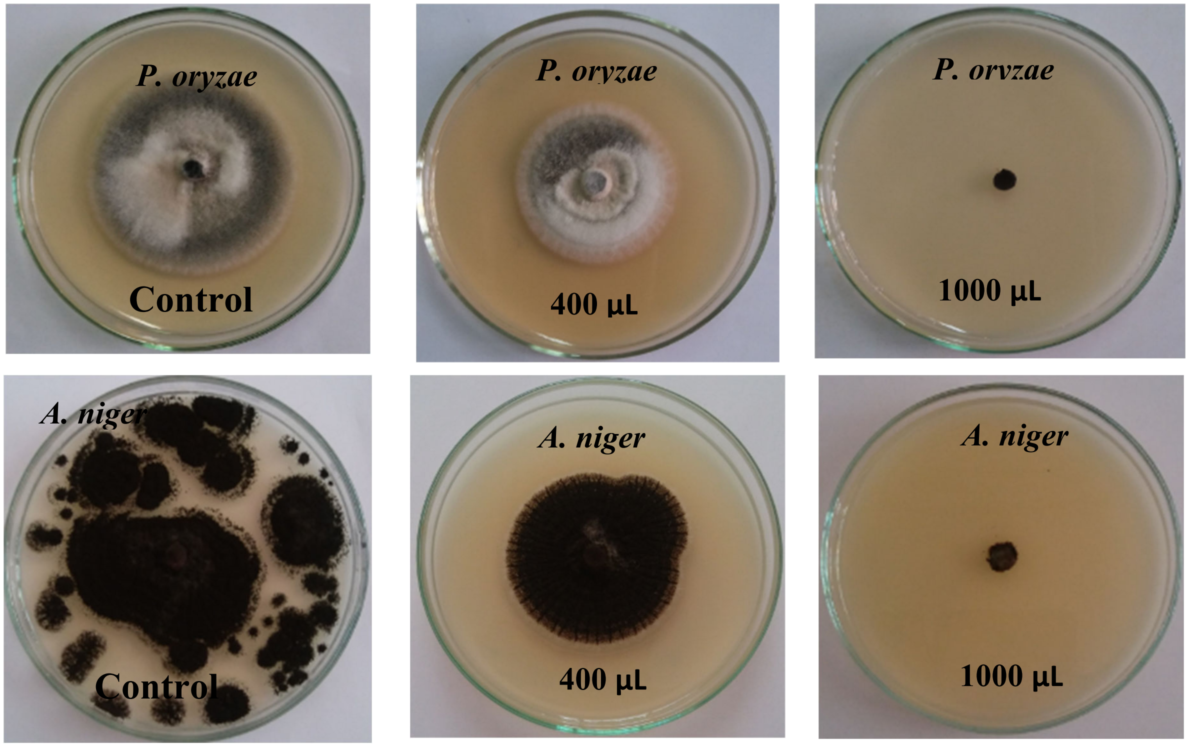

Figure S1. The effect of different concentrations of the *G. persica* extract (control, 400, and 1000 µL) on the mycelial growth of *P. oryzae* and *A. niger*.

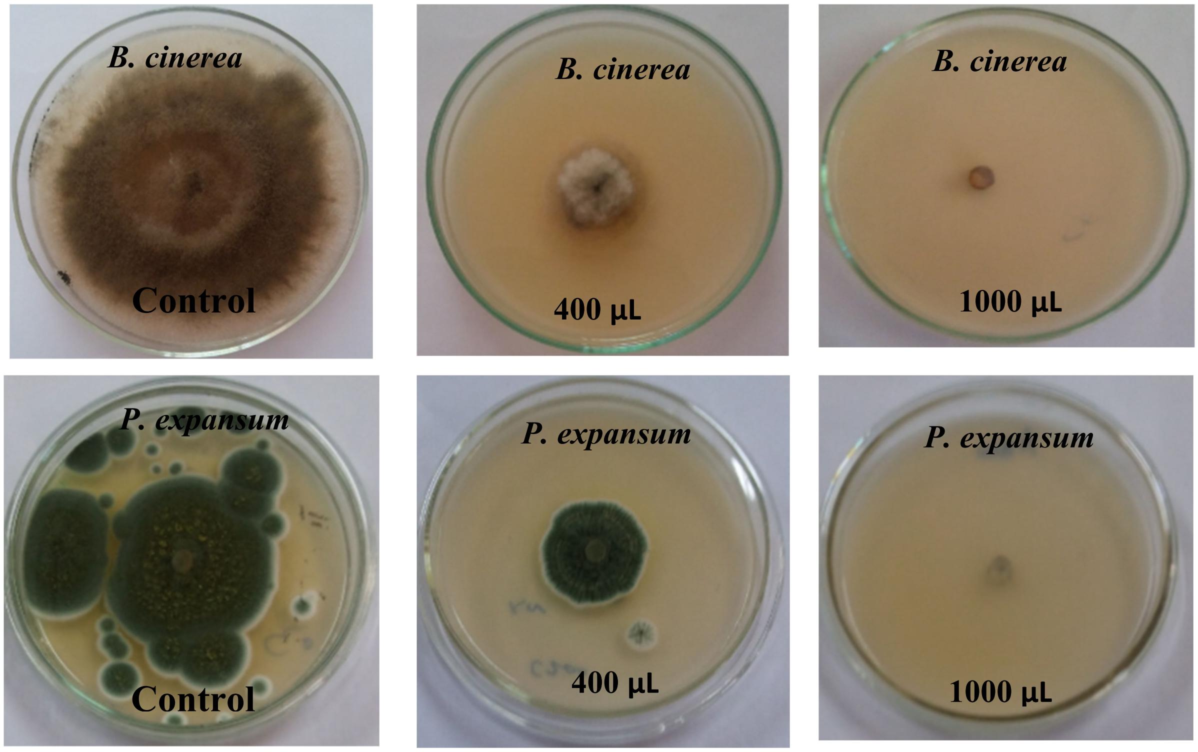

Figure S2. The effect of different concentrations of the *G. persica* extract (control, 400, and 1000 µL) on the mycelial growth of *B. cinerea* and *P. expansum*.
